# Simulations of pH and thermal effects on SARS-CoV-2 spike glycoprotein

**DOI:** 10.3389/fmolb.2025.1545041

**Published:** 2025-02-11

**Authors:** Ziyuan Niu, Georgios Kementzidis, Miriam Rafailovich, Marcia Simon, Evangelos Papadopoulos, Bertal H. Aktas, Yuefan Deng

**Affiliations:** ^1^ Department of Applied Mathematics and Statistics, Stony Brook University, Stony Brook, NY, United States; ^2^ Department of Materials Science and Chemical Engineering, Stony Brook University, Stony Brook, NY, United States; ^3^ Department of Oral Biology and Pathology, Stony Brook University, Stony Brook, NY, United States; ^4^ Division of Hematology, Brigham and Women’s Hospital, Harvard Medical School, Boston, MA, United States

**Keywords:** SARS-CoV-2, spike protein, AAMD, pH solvent, thermal conditions

## Abstract

We performed triplicate and long-time all-atom molecular dynamics simulations to investigate the structures and dynamics of the SARS-CoV-2 spike glycoprotein (S-protein) for a broad range of pH = 1 through 11 and temperatures of 3°C through 75°C. This study elucidates the complex interplay between pH and thermal effects on S-protein structures, with implications for its behavior under diverse conditions, and identifies the RBD as a primary region of the structural deviations. We found: 1) Structural deviations in the S-protein backbone at pH = 1 are 210% greater than those at pH = 7 at 75°C, with most of the deviations appearing in the receptor-binding domain (RBD). Smaller structural changes are observed at pH = 3 and 11. 2) The pH and thermal conditions impact on the protein structures: substantial acidic and basic conditions expand the protein’s solvent exposure, while high heat contracts. This effect is primarily pH-driven at extreme acidity and thermo-driven at moderate pH. 3) The Gibbs free energy landscape reveals that pH as the main driver of structural changes. 4) The parametrized methods enable the predictions of the S-protein properties at any reasonable pH and thermal conditions without explicit MD simulations.

## 1 Introduction

Environmental factors such as pH (Talley and Alexov, 2010) and thermal conditions (Tilton et al., 1992) play a crucial role in the stability of virus and protein structures. Strong acids and bases are frequently used for antiviral sterilization (Rowan et al., 2021), while research from various *in vitro* studies (Sturman et al., 1990; Darnell et al., 2004; Chu et al., 2006) indicates that viruses can maintain their infectivity across a broad spectrum of pH conditions. These findings underscore the need to examine the structural stability of viral proteins, like the SARS-CoV-2 spike protein, under extreme pH conditions. Moreover, the pH within early endosomes starts at pH = 6.3 and gradually drops to pH < 5 within lysosomes (Chen and Geiger, 2020). The gastric acid and the gastrointestinal tract are approximately pH = 1.5 to 7.4 [6, 7]. By examining the S-protein at pH levels as low as 1, we aim to capture the protein’s behavior under extreme conditions that mimic acidic environments.

Moreover, exposure to heat is a widely adopted strategy for family antiviral disinfection, even though certain viruses can withstand extreme temperatures, both high and low (Leclercq et al., 2014; Van Doremalen et al., 2020). Prior *in vitro* research (Lamarre and Talbot, 1989; Chan et al., 2011; Islam et al., 2020) has demonstrated significant couplings of the pH and thermal conditions on the coronavirus properties. Revealing the intersection relation of SARS-CoV-2 across various pH and thermal conditions is crucial for mitigating virus transmission and comprehending viral biological functions. Recent research indicates that SARS-CoV-2 maintains stability at room temperature from pH = 3 through pH = 10 (Chin et al., 2020). The SARS-CoV-2 retains its stability for up to 2 weeks at 4°C and 24 h at room temperature but becomes inactive in 5 min at 70°C (Chin et al., 2020). However, the detailed pH and thermal coupling effect on SARS-CoV-2 requires further investigation.

Numerous works (Kandzia et al., 2019; Ostrowska et al., 2019; Leonard et al., 2021; Zhang and Huang, 2021) have adopted computational simulations to enable detailed examination of complex systems safely and inexpensively. The *in silico* studies and conventional laboratory experiments enhance mutually in gaining temporal and spatial resolutions and deep scientific insights. For example, the widely adopted all atomic MD reveals the protein structure details at the atomic scale that laboratory experiments miss (Frances-Monerris et al., 2020; Niu et al., 2022). Thus, the development of accurate computational models is crucial for understanding key factors that influence viral activity. Limited by the prohibitive computational costs of simulating an entire virus using all atomic MD, we focus on the virus’ S-protein that is, albeit much smaller, an essential virus virulence determinant. The S-protein, situated on the coronavirus envelope, attaches to the host cell angiotensin-converting enzyme 2 (ACE2) during infection (He et al., 2020).

The structural alternations to the S-protein resulting from any conditions including pH or thermal effects may alter the infectivity of the hosting virus. Various *in silico* models suggest that the S-protein remains stable in the 0°C–30°C range, yet mixed opinions persist at higher temperatures, particularly, at 40°C–60°C (Rath and Kumar, 2020; Marti et al., 2021; Khan et al., 2022; Niu et al., 2022). The RBDs of the S-protein exhibit the greatest stability from pH = 6 to pH = 9 (Xie et al., 2022). Beyond their individual effects, the complex coupling effects at pH and thermal conditions are also evident in the structure and dynamics of S-protein. For instance, lowering the pH from 7.4 to 6.0 has been shown to reduce the thermal sensitivity of the S-protein structure (Edwards et al., 2021; Warwicker, 2021). Our study aims to uncover more intricate details in pivotal pH and thermal conditions.

The stability and structural conformations of the RBD are key factors in determining the transmissibility and infectivity of SARS-CoV-2. The RBD demonstrates a higher affinity for ACE2 binding compared to other coronaviruses (Costa et al., 2020; Shang et al., 2020b). The precise details of MD simulation allow existing studies to capture the contributions of single residues on the RBD to the interactions with ACE2 (Pipito et al., 2022). The S-protein has two main conformations: a closed form, with all its RBDs down, and an open form, where at least one RBD is up (Walls et al., 2020; Wrapp et al., 2020; Yan et al., 2021). Insights from recent studies (Ke et al., 2020; Turonova et al., 2020) reveal that the ratio of these conformations varies with external conditions. Furthermore, the S-proteins elude antibody neutralization by fluctuating between the open and closed states (Gur et al., 2020; Zhou et al., 2020). The closed S-protein plays a crucial role in eluding the surveillance of the host immune system, thereby enhancing the spread of the virus (Frances-Monerris et al., 2020; Shang et al., 2020a). Both *in silico* (Cia et al., 2022) and *in vitro* (Ke et al., 2020; Juraszek et al., 2021) studies indicate that the S-protein trimer predominantly remains in a closed conformation before binding to ACE2 and initiating viral entry into host cells. Therefore, our study focuses on the closed states of the S-protein, 6VXX.pdb (Walls et al., 2020) which crucial for the viral immune evasion and persistence.

In consideration of their physiological and practical significance, we perform MD simulations for pH = 1 (extreme acid), 3 (acid kitchen cleaner), 5 (lysosomes), 7 (body pH), 9 (bar soap) and 11 (household ammonia). At each pH, temperatures were set at 3°C (cold supply chain), 20°C (room temperature), 37°C (body temperature), 56°C (critical temperature) and 75°C (high temperature). The simulated time for experiment reached 450 ns, a significant increase over the time scales attained in other studies with similar system sizes (Malaspina and Faraudo, 2020; Rath and Kumar, 2020; Marti et al., 2021; Coutinho et al., 2022; Khan et al., 2022; Sahihi and Faraudo, 2022). This extended duration brings the S-protein closer to equilibrium, offering a solid foundation for our analyses. A detailed comparative table summarizing all relevant information is provided in our previous work (Niu et al., 2024). We analyzed the of S-protein hierarchically (at protein, domain and residue bases) under 30 different conditions. The protein-based analysis reveals overall conformational changes, whereas examining them at the residue level offers more detailed insights into individual residue alterations, aiding in identifying factors that influence overall changes. Given the critical role of the RBD in the S-protein, we have also conducted separate analyses of RBD alterations across various environments which provides a deeper understanding of the S-protein’s behavior. Furthermore, our parametrized root mean squared deviation (RMSD) method leverages the 30 sets of discrete MD simulation data to forecast changes in the outcome distribution across a broad and continuous spectrum of pH and thermal conditions.

## 2 Generation and analysis of trajectories

### 2.1 The *in silico* experiments

Based on recent *in vitro* and *in silico* studies (Chin et al., 2020; Xie et al., 2022), we design simulations for six pH levels and five temperatures, resulting at 30 unique conditions, with triplicates conducted for each. The choice of pH = 1 represents an extremely acidic environment, allowing us to examine potential structural changes or denaturation in the S-protein under highly acidic conditions. Examining the limits of S-protein stability at such extreme acidity provides valuable insights into its structural resilience and mechanisms of denaturation. By comparison, pH = 3 and 11, while less extreme, are included to assess whether critical points exist where notable structural changes occur. The mild pH = 5, 7, and 9 conditions permit evaluations of the S-protein stability at conditions that resemble the human body physiological environment.

The 3°C should be tested as it represents the typical cold storage conditions for biological samples and vaccines. Understanding the structural stability of the SARS-CoV-2 spike protein at this temperature is crucial for ensuring the S-protein minimal conformational changes during storage and transport. At the room temperature of 20°C, the S-protein should be included to evaluate the protein’s stability under standard environmental conditions, which are common in laboratory experiments or during short-term handling. At the normal human body temperature of 37°C, we could understand the functions during infection. The higher temperature of 56°C is considered a threshold for many viruses, SARS-CoV-2 included, to denature within 30 min. Therefore, it is essential to test this critical point to assess the potential for denature. Finally, the 75°C should be tested as it represents an extreme thermal condition, providing information on the denaturation dynamics.

The MD simulations adapt the open-source GROMACS (Abraham et al., 2015) that was coupled with the CHARMM36 force field (Brooks et al., 2009; Best et al., 2012). The initial structure of the S-protein is retrieved from the Protein Data Bank (6VXX.pdb) (Walls et al., 2020), with missing loops in the 6VXX structure reconstructed using Robetta (Baek et al., 2021). The S-protein, comprising 1273 residues per chain, is placed in the explicit solvent. We employ the SPC/E water model, known for its ability to represent solvent accurately, capturing the critical interactions between the protein and water (Mark and Nilsson, 2001; Takemura and Kitao, 2007), with an optimal balance between computational efficiency and accuracy. The simulation box, sized at 21 × 21 × 21 nm³, is subject to periodic boundary conditions in all three Cartesian dimensions, and the density of the system is 1.01 g/ $cm^{3}$ .

In all MD simulations, the energy minimization process was conducted using the steepest descent method. The system equilibration was achieved through both canonical (NVT) and Parrinello-Rahman pressure coupling (NPT) with a 2 fs time step size, and the production runs were maintained in the NPT ensemble. The triplicate experiments are performed at each condition to ensure the accuracy and reproducible of the MD simulations. Each triplicated simulation was initiated by sampling the initial velocities of all atoms in the system according to Maxwell-Boltzmann distribution. For all 90 simulations (3 replicates across 30 unique conditions), the simulation time is extended to 450 ns to ensure sufficient sampling of the S-protein’s conformational dynamics under each condition. On the shared AiMOS supercomputer (Hanson, 2019) configured with IBM POWER9 processors and NVIDIA Volta V100 GPUs, we perform our experiments on a sub-partition of 4 nodes with a running speed of approximately 50 ns/day.

Conventional pH MD simulations may introduce potential biases because the charge states of titratable residues are kept constant. Although constant pH MD simulations can dynamically update these charge states, they are computationally intensive (Buslaev et al., 2022), especially when multiple titratable sites interact both electrostatically and dynamically. Achieving effective sampling without a loss in computational speed poses an additional challenge, particularly for large proteins with hundreds or thousands of titratable sites undergoing major conformational changes (de Oliveira et al., 2022; Lasham et al., 2024). Therefore, we choose to calculate the pKa values for all titratable residues in the S-protein and perform MD simulations with fixed protonation states, making it feasible to study proteins of this scale efficiently.

Empirical computational tools like PROPKA3 (Olsson et al., 2011) can accurately predict the pKa values of ionizable groups within proteins, even when hundreds of residues are involved. We control the protonation and deprotonation states of titratable residues, as estimated by the pKa predictor PROPKA3 (Olsson et al., 2011). To achieve charge neutrality, Cl⁻ and Na⁺ ions are added to acidic and basic solvents, enhancing the accuracy of the protonation states (Chen and Shen, 2014). Detailed information on protein characteristics, including the number of atoms in the protein, the number of atoms in water, and the protein’s net charge, is available in a previous publication (Niu et al., 2024). We maintain these parameters consistent with the previous study to ensure direct comparability and control across results.

### 2.2 Measurements and analysis of protein properties

The S-protein is organized as a homotrimer, with each of its three chains consisting of 1273 amino acids intertwined. Each chain includes three main regions: a signal peptide (residues 1–13), an S1 domain (S1D, residues 14–685) and an S2 domain (S2D, residues 686–1273). The S1 and S2 domains of the S-protein play a crucial role in infecting a host cell, with S1D binding to the host cell receptor (He et al., 2020) and largely composed of beta-sheets (Walls et al., 2016), includes the N-terminal domain (NTD) and the receptor-binding domain (RBD) (Xia et al., 2020). The S2D fusing with the virus (He et al., 2020) and primarily containing α-helices that span the membrane (Walls et al., 2016).

Data analysis reflects averages from three independent experiments. To reveal the structural changes in the S-protein, we conduct hierarchical analyses at the protein, domain and residue levels. For the full-length protein, we evaluate backbone RMSD, radius of gyration (Rg), solvent accessible surface area (SASA), interface area between chains, and the number of hydrogen bonds (H bonds) formed between the protein and water molecules (P-W) as well as inter-mainchain H bonds, collectively reflecting the overall structural changes in the S-protein. Additionally, we examine the Gibbs free energy (GFE) landscape and secondary structure alterations.

At the domain level, our analysis includes RMSD for specific domains such as the NTD, RBD and S2D. For residue level insights, we measure the root mean squared fluctuation (RMSF) for each individual residue.

#### 2.2.1 Gibbs free energy landscape

The GFE landscape is calculated as

$$∆G\left(x_{1},x_{2}\right)=-k_{B}T\ln P\left(x_{1},x_{2}\right)$$

where $k_{B}$ is the Boltzmann constant, *T* denotes temperature, and $P\left(x_{1},x_{2}\right)$ is the normalized joint probability for the first two principal components (PC1 and PC2) which capture most structural variation in the carbon alpha trajectory. The covariance matrix for the carbon alpha coordinates is computed using trajectory data from the last 200 ns, with alignment to the first frame to mitigate rotational and translational effects in the simulation. By diagonalizing the covariance matrix, the resulting eigenvalues and eigenvectors allowed for projection onto PC1 and PC2, enabling the creation of a two-dimensional free energy landscape plot of the conformational space (Maisuradze and Leitner, 2007).

#### 2.2.2 Parametrized RMSD

Non-linear least square analysis is applied to fit the RMSD data for the full protein, NTD, RBD and S2D for a range of pH and thermal conditions. This allows us to fit these data to obtain a function of the RMSD’s in two variables--pH and temperature--and several parameters. Many function forms can be candidates and, multiple trials and insight of chemistry lead us to the following fitting function for all four measures:

$$\text{RMSD}\left(T,p^{*}\right)=R_{0}*\left(1+\alpha_{T}T\right)*\left(e^{{-\beta }_{a}p^{*}}+e^{\beta_{b}p^{*}}\right)$$

where *T* represents temperature and p*=pH−7 . The coefficients $R_{0},\alpha_{T},\beta_{a}$ and $\beta_{b}$ are free parameters fitted to the data, capturing the effects of pH and thermal conditions on the RMSD. More specifically, the $\beta_{a}$ represents the coefficient dominant under acidic conditions where p*<0 , while $\beta_{b}$ applies to basic conditions.

Thermal effects are modeled linearly because no extreme structural shifts were observed within the ns time scales for the tested temperature range. This model, allowing us to assess the individual effects, and their couplings, at the pH and thermal conditions, offers predictive insights for protein structures at the untested conditions.

The pH dependence is modeled by two exponential functions to depict the asymmetrical pH effects of the acidic and basic conditions on the protein. This form with the two fitting parameters $\beta_{a}$ and $\beta_{b}$ is flexible and sufficient to express such changes. From a chemical perspective, the relationship between pH and RMSD builds on the hydrogen ion concentration and its influence on protein conformation. RMSD measures structural deviations, which we relate to pH via p*=pH−7 . Here, pH reflects the activity of hydrogen ions, expressed as:

$$\text{pH}\approx -\log_{10}\left[H^{+}\right]=-\frac{\ln\left[H^{+}\right]}{\ln10}$$

To bridge the conceptual gap between pH and structural deviations we incorporate p* and expand our chosen function:

$$e^{{-\beta }_{a}p*}+e^{\beta_{b}p*}\approx e^{{-\beta }_{a}\left(-\frac{\ln\left[H^{+}\right]}{\ln10}-7\right)}+e^{\beta_{b}\left(-\frac{\ln\left[H^{+}\right]}{\ln10}-7\right)}=e^{{7\beta }_{a}}\,{\left(\left[H^{+}\right]\right)}^{\frac{\beta_{a}}{\ln10}}+e^{{-7\beta }_{b}}\,{\left(\frac{1}{\left[H^{+}\right]}\right)}^{-\frac{\beta_{b}}{\ln10}}$$

The fitting parameters $\beta_{a}$ and $\beta_{b}$ express the effect of the concentrations of hydrogen ions on the molecule’s configuration, going deeper into the causes of the changes in the structures of the S-protein.

## 3 Results

During MD simulations, the raw coordinates for all atoms were collected at 0.1 ns intervals and aligned by the protein backbone at the center of the box. We evaluate the conformational stability of the S-protein across these 30 conditions, aiming to establish a foundational understanding of its structural dynamics at varying pH and thermal conditions.

### 3.1 The whole protein

Our protein-based analyses focus on residues 1–1162, which cover the bulbous head and part of the stalk region. This selection excludes the distal portion of the stalk to reduce the impact of its considerable fluctuations on our assessment of the S-protein’s stability. The mean values from 250 ns to 450 ns, along with the standard errors from the triplicate simulations, are provided in Supplementary Table S1 for the protein analyses. The time series data for the entire simulation period are shown in Supplementary Figure S1.

**FIGURE 1:**
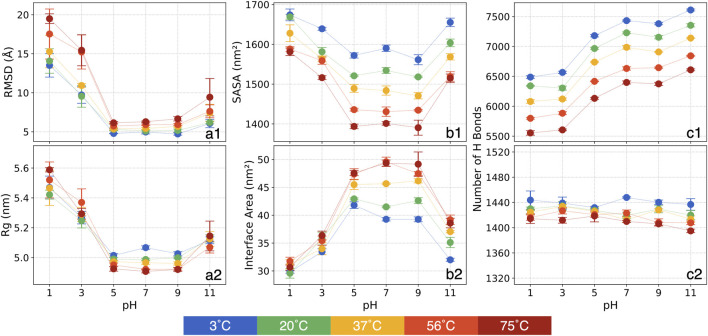
The means and standard errors for various structural metrics: (a1) RMSD, (a2) Rg, (b1) SASA, (b2) chain-chain interface area, (c1) the number of P-W H bonds and (c2) the number of inter-mainchain H bonds.

The RMSDs: We calculate RMSD by averaging the backbone atom coordinates at each timestep relative to their positions in the initial structure. The mean of RMSD values over the last 200 ns and the standard errors from the triplicate simulations are presented in Figure 1a1.

In general, at a given temperature, the RMSD analysis reveals a high sensitivity at more acidic conditions of pH = 1 and 3. For pH = 1, in contrast to pH = 7 at the same temperature, RMSD increased by 170% at 3°C and by 210% at 75°C. At pH = 3, RMSD again separates into two temperature-dependent groups. For temperatures below 37°C, RMSD increases by roughly 90%–110%, while for temperatures above 56°C, it rises by approximately 150%. Conversely, at pH = 5 and 9, RMSD deviates minimally from that of pH = 7 regardless of temperatures, indicating relative stability. At pH = 11, RMSD shows less variation compared to acidic conditions, with the largest change occurring at 75°C, where RMSD increases by around 50%.

At a given pH condition, RMSD increases with temperature. This increase remains modest (within 2 Å) for pH = 5, 7, and 9. However, at pH = 1 and 3, the thermal effect on the S-protein intensifies considerably. Notably, at pH = 1, RMSD is 13.5 Å at 3°C and rises steadily with temperature, reaching 19.5 Å at 75°C. At pH = 3, RMSD exhibits a distinct separation into two groups with increasing temperature. For temperatures between 3°C and 37°C, RMSD is centered around 9.6 Å, but it escalates to 15.3 Å at 56°C and 75°C. At pH = 11, RMSD also rises with temperature but to a lesser extent than at acidic conditions, increasing from 6.1 Å to 9.4 Å between 3°C and 75°C.

The considerable RMSD variations at pH = 3 with rising temperatures highlight three distinct structural states for the 30 conditions: 1) RMSD as high as 13.5 Å is observed at pH = 1 and all temperatures and at pH = 3 for relatively higher temperatures, showing substantial structural deviations; 2) Intermediate RMSD at around 10 Å is observed at low temperatures for pH = 3 and at 75°C for pH = 11, indicating the S-protein undergoing a transitional phase; and 3) Low RMSD at around 5.5 Å is observed at pH = 5 through pH = 11 at all tested thermal conditions, revealing minimal structural deviations.

The Rg represents how atoms are distributed relative to the protein’s center of mass, providing valuable insights into the protein’s overall size and structural configuration. A lower Rg value reflects a more compact protein structure, while a higher Rg indicates a more extended form. Shifts in Rg serve as important markers for alterations in the protein’s tertiary structure. The mean of Rg values and standard errors over the last 200 ns of the triplicate simulations are shown in Figure 1a2.

Our analysis reveals that, at a given temperature, Rg increases by approximately 10% at pH = 1 and 5% at pH = 3 compared to pH = 7, indicating a more expanded structure under highly acidic conditions. This expansion is likely due to the protonation of amino acid side chains, which disrupts the electrostatic interactions, leading to a looser protein structure. At basic conditions at pH = 11, Rg increases only slightly (around 2%), indicating a minor expansion.

Thermal effects on Rg, however, are less visible at any pH values. Overall, Rg decreases slightly (about 2%) with increasing temperature from 3°C to 75°C at each pH, suggesting a trend toward a more compact structure as temperature rises. This trend hints at a tendency of the S-protein to adopt a more condensed conformation at increasing temperatures, a result corroborated in subsequent analyses of SASA and interface area.

These observations suggest that the S-protein remains relatively compact at neutral and mildly basic conditions, while extreme acidic conditions promote expansion. Additionally, the protein tends to remain more extended at lower temperatures. Thus, our Rg analysis highlights distinct effects at pH and thermal conditions on the S-protein’s conformation.

The SASA analysis offers more insights into the folding patterns and stability of the S-protein, particularly regarding its overall shape, whether more extended or contracted. The mean of SASA values over the final 200 ns, along with the standard errors from the triplicate simulations, are presented in Figure 1b1.

At a constant temperature, SASA increases at extreme pH conditions. At pH = 1, SASA increases by approximately 85 nm^2^ at 3°C, with this upward trend becoming more pronounced as temperature rises. By 75°C, SASA shows an increase of 180 nm^2^ at pH = 1 compared to pH = 7. Similarly, pH = 3 and 11 display increased SASA relative to pH = 7, although the increase is less than at pH = 1. For pH = 5 and 9, SASA shows only minor deviations from that of pH = 7. The elevated SASA at pH = 1, 3 and 11 suggest greater structural deviation in specific domains of the S-protein, indicating an expansion relative to its structure at pH = 7. Further analyses will offer more detailed insights into the specific structural changes in these domains. When comparing different thermal conditions at a constant pH, SASA decreases as temperature increases. The SASA reduction is more pronounced, approximately 180 nm^2^, within the moderate pH range, whereas it decreases by around 130 nm^2^ at pH = 3 and 11. At pH = 1, SASA decreases by only 93 nm^2^, indicating that thermal effects are impeded by the extreme pH conditions.

Overall, the analysis reveals two primary trends: SASA decreases with increasing temperature and tends to be higher under strongly acidic or basic conditions. This suggests that extreme pH promotes an extension of the S-protein, while high temperature promotes a more contracted state. Additionally, the results show couplings where the thermal effects diminish at more acidic conditions.

The chain-chain interface area represents the regions where the S-protein chains make contact and interact, playing a crucial role in stabilizing the quaternary structure of the trimeric S-proteins. The average interface areas between chains were calculated using the PDBePISA [61] server and are displayed in Figure 1b2.

In our analysis, the interface area decreases substantially under acidic and basic pH conditions at the same thermal conditions. While pH = 5 and 9 show relatively stable values close to those observed at pH = 7, pH = 11 exhibits a notable reduction of approximately 8 nm^2^ across all thermal conditions. A clear trend appears in acidic conditions: the interface area decreases more significantly at higher temperatures. Specifically, at pH = 1, the interface area averages around 30 nm^2^ across all thermal conditions. Compared to pH = 7, the interface area at 3°C decreases by 10 nm^2^, but this difference grows to 18 nm^2^ at 75°C. A similar trend, though less pronounced, is observed at pH = 3.

Interestingly, at a constant pH, the interface area tends to increase as temperature rises, especially at pH > 5. For example, at pH = 7, the interface area increases by around 10.3 nm^2^ from 3°C to 75°C, and at pH = 9 and 11, it increases by approximately 7.5 nm^2^. At acidic conditions, however, this increase is more moderate: at pH = 5, it only increases by about 5.6 nm^2^, and at pH = 1 and 3, the increase is less than 2 nm^2^.

These findings collectively suggest that interchain interactions weaken under strongly acidic and basic pH, particularly at pH = 1, 3, and 11, which may result in structural changes or partial separation of the trimer into individual chains. Additionally, heating tends to increase the interface area, indicating a compaction effect as confirmed by SASA measurements, with chains moving closer together. An interesting intersectional effect is observed: at extreme pH conditions, pH appears to dominate, causing a smaller increase in interface area even at higher temperatures. In contrast, at moderate pH conditions, thermal effects dominate the variations of the interface area.

The H bonds: Experimental studies indicate that H bonds play a critical role in protein stability (Pace et al., 2014), rooted in the significance of H bonds in the shaping of the tertiary structure. In our analysis, H bonds were counted based on cutoff values for the angle and distance between donor and acceptor atoms. The mean of H bond counts for the final 200 ns and the standard errors from the triplicate simulations are shown in Figures 1c1, c2 for P-W and inter-mainchain H bonds, respectively.

At a constant temperature, the number of P-W H bonds tends to decrease at acidic conditions and increase at basic conditions. Compared to pH = 7, at pH = 1 and 3, around 900 P-W H bonds are broken. At pH = 5, approximately 200 P-W H bonds break. At pH = 9, the number of P-W H bond shows no noticeable change, whereas at pH = 11, around 200 additional P-W H bonds formed. Interestingly, while pH = 1 and 3 show a significant decrease in P-W H bonds relative to pH = 7, there is an increase in SASA at low pH. This indicates that as the protein unfolds, hydrophobic residues typically buried in the core may become more exposed, leading to increased SASA but competing with water molecules in forming the P-W H bond. Conversely, at pH = 11, the formation of additional P-W H bonds aligns with increased SASA, suggesting that more hydrophilic residues are interacting with the solvent.

When comparing different thermal conditions at a constant pH, a clear linear trend can be found, approximately 1,000 P-W H bonds are broken as it heats up from 3°C to 75°C. Compared to SASA, both measures decrease with increasing temperature, indicating a distinct structural response likely due to high temperatures causing a contraction of the protein. An interesting result in the P-W H bond analysis is that, unlike with other metrics, pH and thermal conditions appear to influence protein structural variations independently.

Across all tested pH values, no significant changes are observed in the counts of inter-mainchain H bonds at any given thermal condition. Within the same pH, as temperature increased from 3°C to 75°C, the inter-mainchain H bonds broken by less than 2%, a minimal reduction. This finding suggests that mainchain interactions remain highly stable, even at extreme pH and high temperatures.

**FIGURE 2:**
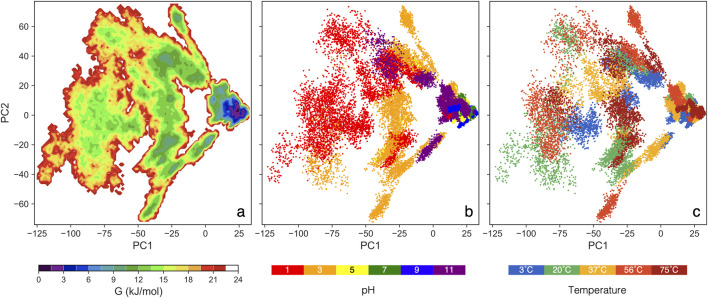
The color maps sowing GFE landscape analysis vs two major principal components: **(A)** colors show free energy, **(B)** colors show pH values for all thermal conditions, and **(C)** colors show thermal conditions for all pH values.

The GFE landscape: It is important to note that the GFE value is not energy measured from first principles but a statistical approximation based on the assumption that the equilibrium distribution of states follows the Boltzmann distribution (Papaleo et al., 2009). Each point on the plot represents a conformational state from the MD simulation, with smaller distances between points indicating structural similarity and denser regions suggesting stable protein conformations. The GFE landscape for our 90 simulations (spanning 30 conditions with triplicates) is presented in Figure 2. Each simulation includes data from 250 ns to 450 ns, concatenated to construct a single comprehensive covariance matrix. PC1 accounts for 42%, while PC2 accounts for 13% of the total variance across all conditions.

In Figure 2B, a clear trend is pH = 5, 7, and 9 cluster closely, aligning with a deep valley in Figure 2A, which indicates consistent and stable protein conformations. In contrast, pH = 3 and 11 form broader, shallower ensembles than the moderate pH range, though still deeper than pH = 1. At pH = 1, the protein spans a large, flat region in Figure 2A, suggesting the most extensive structural variation. The deep energy valleys at moderate pH suggest that the S-protein favors stable conformations within this range, with thermal condition having little impact on structural stability (Chin et al., 2020; Xie et al., 2022). These GFE landscapes demonstrate notable pH-dependent variations, highlighting pH as a key factor influencing the conformational dynamics of the S-protein.

Spatial distribution within the landscape also provides insights into structural similarity, as the relative positions and distances between ensembles reflect the degree of geometric resemblance (Becker, 1997). Trajectories at pH = 5, 7, and 9 project onto similar PC1 and PC2 values, clustering closely across all thermal conditions, which suggests that the protein adopts similar conformations within this moderate pH range. Conversely, the trajectories at pH = 1 are significantly separated from the moderate pH cluster, covering a broader range of conformations. Notably, pH = 3 and 11 occupy an intermediate region, serving as a transition between the extreme pH = 1 and the moderate pH range. In Figure 2C, no distinct trend emerges, reinforcing that pH conditions, rather thermal, are the primary driver of structural changes in the S-protein.

The secondary structures: Define Secondary Structure of Proteins (DSSP) (Kabsch and Sander, 1983; Touw et al., 2015) provides quantitative insights by determining the number of residues involved in various secondary structural elements, offering a perspective on the structural features that contribute to protein stability and folding. Supplementary Table S2 presents the DSSP analysis results across our 30 simulation conditions. The Secondary Structure (SS) column shows the combined percentages of structural elements such as α-helix, β-sheet, β-bridge, 3_10_-helix, π-helix and turns. These results suggest that the secondary structure of the S-protein remains relatively stable, maintaining around 60% integrity across diverse pH and thermal conditions, including the extreme combination of pH = 1°C and 75°C.

This stability suggests that the secondary structure of the protein is well-maintained under a range of pH and thermal conditions, at least for the initial 450 ns of the simulation within our conditions. Furthermore, the DSSP and inter-mainchain H bond analyses across the 30 conditions consistently show secondary structural stability. We hypothesize that, in longer simulations, the secondary structure may become destabilized under more extreme conditions, such as highly acidic or high-temperature environments, like pH = 1°C and 75°C.

### 3.2 The domain-based analysis

**FIGURE 3:**
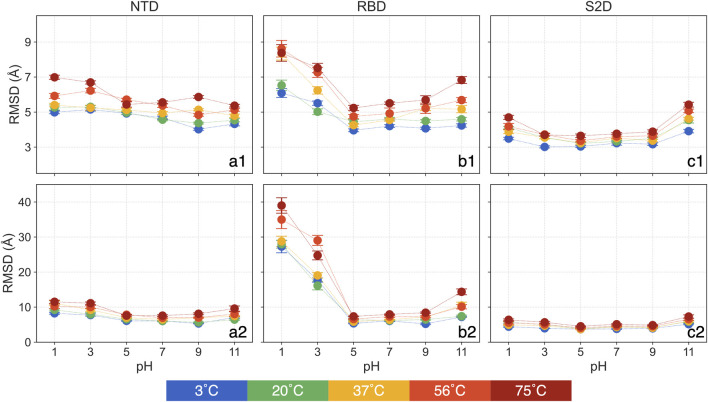
The means and standard errors for domain-based RMSD: (a1) NTD aligned with itself, (a2) NTD aligned with the whole protein, (b1) RBD aligned with itself, (b2) RBD aligned with the whole protein, (c1) S2D aligned with itself and (c2) S2D aligned with the whole protein.

In examining domain-specific conformational changes, specifically in the NTD, RBD and S2D, we focus on the backbone RMSD to assess their relative stability and flexibility. The RMSD is calculated by aligning each domain both to itself and to the entire protein, allowing us to interpret two aspects of structural change: 1) The RMSD aligned to the domain itself reveals internal structural deviations, capturing changes independent of backbone translational and rotational movement in the simulation. 2) The RMSD aligned with the whole protein shows how each domain shifts relative to the entire structure, highlighting changes driven by domain translation and rotation. This targeted approach allows us to assess each domain’s potential to act as a pH or temperature sensor, offering insights into the functional implications of structural adaptation. All backbone RMSD analyses in this section focus on the mean over the 250 ns–450 ns, with standard errors from the triplicate simulations shown in Figure 3 and Supplementary Table S3. In Figure 3, the first row presents RMSD values aligned by each domain, while the second row shows RMSD values aligned by the whole protein. The time series data for the entire simulation period are shown in Supplementary Figure S2.

The RMSD of the NTD, aligned with itself (Figure 3a1), does not display any clear trend across different pH values. Only at 75°C, the NTD RMSD show approximately a 25% increase for pH = 1 and 3 compared to pH = 7. At same pH, the NTD RMSD showing a slightly increase as it heats up from 3°C to 75°C, for example, it increases less than 17% (0.8 Å) at pH = 7. The NTD RMSD, at pH = 5 and 9 and aligned with the entire protein (Figure 3a2), shows no significant deviations from that of pH = 7, regardless of temperatures. However, at both acidic and basic conditions, NTD RMSD increases, with greater sensitivity observed in acidic environments. For instance, at pH = 1 and 3, RMSD is as high as 11.5 Å at 75°C, while at pH = 11, it is 9.6 Å compared to 7.6 Å at pH = 7. At a constant pH, the NTD RMSD slightly increases with rising temperature. In summary, the NTD remains relatively stable at the tested pH and thermal conditions with minor changes at higher temperatures. However, at acidic conditions, the NTD is more prone to rotation and translation.

The RMSD of the RBD, aligned with itself (Figure 3b1), reveals a clear intersection between pH and thermal effects. At low temperatures, only acidic conditions significantly affect the RBD, but as temperature increases, basic conditions (pH = 11) also begin to impact. Specifically, at 3°C and 20°C, RBD RMSD at pH = 11 shows no significant difference from pH = 7. However, at 37°C and 56°C, RBD RMSD at pH = 11 rises by about 15% compared to pH = 7, and by 24% at 75°C. The RBD RMSD remains stable for pH = 5 and 9 across all thermal conditions. At pH = 1, RBD RMSD increases by approximately 43% to around 6.3 Å compared to around 4.4 Å at pH = 7 at 3°C and 20°C, reaching 8.3 Å at 37°C but showing no additional change at higher temperatures.

For the RBD RMSD aligned with the whole protein (Figure 3b2), pH = 5 and 9 again show no significant deviations from pH = 7 across thermal conditions. The RBD RMSD, aligned with the protein, follows a similar trend to the self-aligned data, clustering into three groups: 7.3 Å at 3°C and 20°C, 10.2 Å at 37°C and 56°C, and 14.4 Å at 75°C. At extremely acidic conditions (pH = 1 and 3), RMSD rises dramatically. At pH = 1, RMSD increases to around 28 Å at 3°C to 37°C and further to 35 Å and 39 Å at 56°C and 75°C, respectively, with a similar but slightly lower pattern at pH = 3.

These results highlight three key observations: 1) Individual structural deviations in RBD are most pronounced at pH = 1 and 3 at any temperatures, showing effects only at high temperatures for pH = 11; 2) Structural changes at pH = 1 peaks at 56°C. 3) The RBD experiences considerable translation and rotation in highly acidic conditions (pH = 1 and 3), a result with possible implications on the binding to the ACE2 receptor and hence the infectivity of the virus at these conditions. This observation aligns with prior findings (Xie et al., 2022).

The RMSD of the S2D, whether aligned with itself (Figure 3c1) or with the entire protein (Figure 3c2), shows a distinct trend compared to the NTD and RBD. At pH = 3, 5, and 9, there are no significant deviations from pH = 7 across all thermal conditions, with only pH = 1 and 11 exhibiting an increase in RMSD relative to pH = 7. Interestingly, this increase is more pronounced at pH = 11, suggesting that the S2D demonstrates a high tolerance to acidic environments.

**FIGURE 4:**
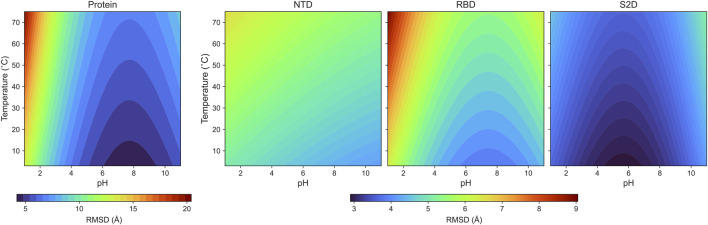
The color maps of the parameterized RMSDs for the protein and its domains: NTD, RBD and S2D.

The parametrized RMSD: Our coefficients are listed in Table 1, along with the coefficient of determination (*R* ^2^), and the results are presented in Figure 4. In this analysis, $\beta_{a}$ represents the dominant coefficient at acidic conditions, while $\beta_{b}$ applies under basic conditions. We observe that for both overall protein RMSD and RBD RMSD, $\beta_{a}>\;\beta_{b}$ , indicating that the structural changes of the S-protein at acidic pH (especially pH = 1 and 3) are more skewed. Conversely, the S2D shows the opposite trend, with $\beta_{b}>\;\beta_{a}$ , consistent with the domain data for pH = 11, which shows greater changes than at acidic conditions like pH = 1. The NTD domain displays much smaller $\beta_{a}$ and $\beta_{b}$ values compared to other domains, and Figure 4 shows a less pronounced parabolic curve for the NTD, indicating minimal influence of pH changes, particularly at basic conditions.

**TABLE 1:** The coefficients for the protein and its individual domains.

	$R_{0}$	$\alpha_{T}$	$\beta_{a}$	$\beta_{b}$	$R^{2}$
$R_{\text{protein}}$	2.20	0.007	0.29	0.20	0.92
$R_{\text{NTD}}$	2.19	0.004	0.06	0.02	0.84
$R_{\text{RBD}}$	1.88	0.006	0.18	0.15	0.90
$R_{S2D}$	1.47	0.003	0.13	0.20	0.90

### 3.3 The residue-based analysis

Finally, our residue-based analysis delves into the behavior of individual amino acids within the S-protein, measuring their specific fluctuations. This level of detail helps us pinpoint particular residues that may be essential to the protein’s overall stability.

**FIGURE 5:**
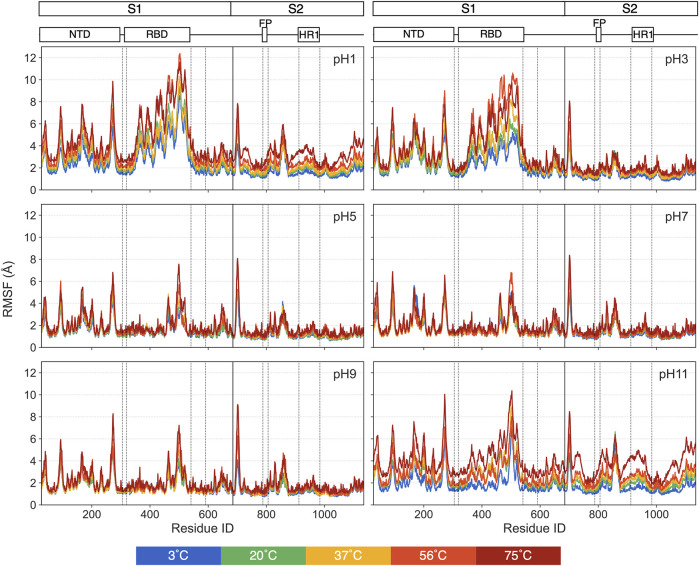
The RMSFs for each pH value, with distinct colors representing different thermal conditions.

**FIGURE 6:**
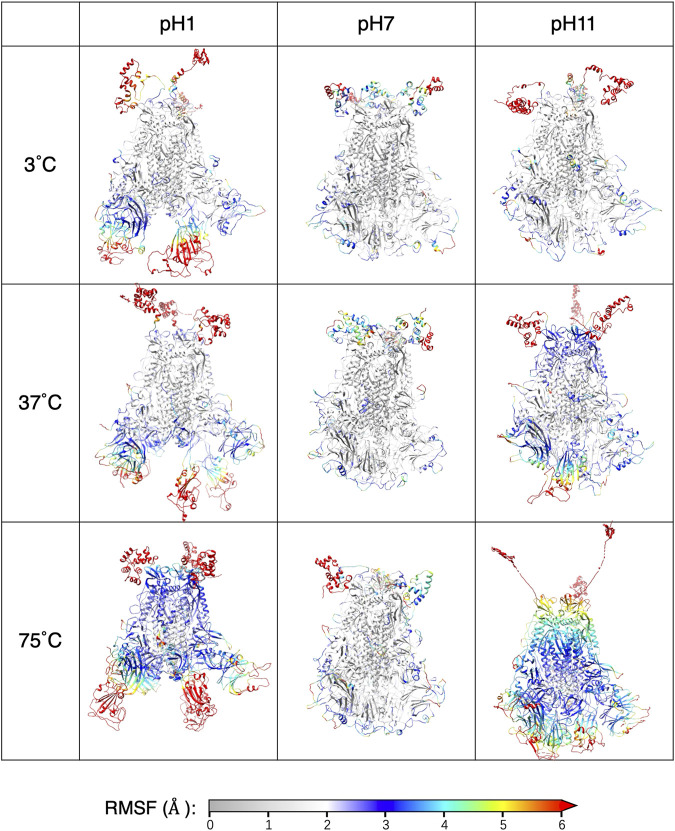
The averaged structure with each residue colored by the RMSF.

For individual residues, we use RMSF to quantify time-averaged fluctuations from their initial positions within a time window of 250 ns–450 ns. The RMSF values for each pH values are shown in Figure 5, with distinct colors representing each thermal condition. Residues in the heptad repeat sequence 2, transmembrane and cytoplasmic tail consistently display high RMSF across all pH and thermal conditions, reflecting their inherent flexibility in the S-protein stalk regions. The Figure 6 displays the S-protein structure averaged over the 250 ns–450 ns trajectory under selected conditions, highlighting the differences between low and high temperatures as well as acidic and basic environments. Residues are color-mapped to reflect their respective RMSF values. A comprehensive structure figure of all 30 conditions is provided in Supplementary Figure S3.

While most residues remain relatively stable, some display significant fluctuations under extreme pH, suggesting these residues may contribute to major structural changes in the S-protein. At moderate pH, even heat has minimal impact on RMSF. At pH = 1, 3 and 11, most of the highly fluctuating residues, with RMSF values exceeding 5 Å, are in the RBD and NTD, with increasing fluctuations at increasing temperatures. At pH = 1 and 11, a few residues in the S2 domain also exhibit unusually high RMSF values compared to pH = 7.

## 4 Discussions

While current experiments show that SARS-CoV-2 maintains high stability between pH = 3 and 10 at room temperature and remains stable for up to 2 weeks at 4°C, it rapidly loses activity within 5 min at 70°C (Chin et al., 2020). However, the specific interactions between pH and thermal effects on the S-protein, and their implications for structural stability, are not fully understood. This study offers an in-depth examination of the structure and dynamics of the SARS-CoV-2 S-protein at pH = 1 through pH = 11 and temperatures 3°C through 75°C. Our findings reveal the individual, and coupling, effects of pH and thermal conditions.

The RMSD analysis reveals three distinct structural states of the SARS-CoV-2 S-protein. The protein remains structurally stable at pH = 5, 7, and 9, consistent with *in vitro* findings on other coronaviruses under similar pH conditions. At pH = 3 below 37°C and at pH = 11 across all thermal conditions, the S-protein exhibits transitional states where domain-specific structural changes begin, such as alterations in the RBD and NTD. At pH = 1 and at high temperatures for pH = 3, substantial structural deviations occur, suggesting that these conditions push the protein beyond its stable state. Despite these conformational changes, the secondary structure of the S-protein remains notably stable across a range of conditions, as confirmed by DSSP and inter-mainchain H-bond analyses, consistent with studies showing secondary structure stability up to temperatures as high as 60°C (Khan et al., 2022).

The Rg, SASA and chain-chain interface area analyses provide additional detail, indicating that high temperatures lead to protein contraction while extreme pH promotes expansion. An intersectional effect emerges, with highly acidic conditions dominate the influence and thermal conditions become more impactful at moderate pH. This interaction suggests that S-protein stability depends on the combined effects of pH and thermal conditions. Acidic conditions weaken interchain interactions, potentially destabilizing the trimeric quaternary structure, as seen in the reduced chain-chain interface area, which may facilitate partial dissociation of the trimer into individual chains.

The domain and residue-based analysis, analyses reveal further insights into these structural changes. The RBD shows significant structural deviations at pH = 1 and 3, including translation and rotation that could affect binding efficiency. While no obvious change can be detected under moderate pH = 5, 7, and 9, consistent with prior observations (Xie et al., 2022) that the S-protein RBD is most stable between pH = 6 and 9. Conversely, the S2D shows increased sensitivity at basic pH = 11. High RMSF values in the RBD and NTD at pH = 1, 3, and 11 suggest local structural instability. The differential responses of RBD and S2D to acidic *versus* basic environments, as seen in the parametrized RMSD analysis, underscore each domain’s unique adaptability to environmental changes.

We recognize the crucial role that glycan groups play in providing structural stability and aiding immune evasion of the S-protein by shielding it and influencing its interactions with host receptors (Casalino et al., 2020; Watanabe et al., 2020). In this study, we exclude glycan groups to concentrate solely on the core protein, a choice made to mitigate computational burden while focusing on our objective: to evaluate the bioactivity and conformational transitions of the S-protein and to examine the correlation between these transitions at varying pH and thermal conditions.

This study expands upon prior research (Niu et al., 2022; Niu et al., 2024) by comprehensively exploring the full range of pH and thermal impacts on the S-protein. Our findings are consistent with recent studies on the stability of coronavirus proteins at various conditions, underscoring the critical roles of pH and temperature in modulating protein structure and stability. Gaining insight into these conformational dynamics is essential for understanding the S-protein’s behavior across different physiological and pathological environments. Additionally, the high computational costs associated with all-atom MD simulations highlight the promise of AI-driven models (Han et al., 2021; Liang et al., 2021; Zhang et al., 2024) in enhancing our ability to simulate complex biological systems. These models offer the potential to alleviate computational demands while effectively capturing the dynamics of the S-protein across a wide range of conditions and states.

## 5 Conclusion

In conclusion, this study elucidates the coupling effects of pH and thermal conditions on the structures and dynamics of the SARS-CoV-2 S-protein. Our analyses reveal that acidic conditions (pH = 1 and 3) lead to significant structural changes, particularly in the RBD and NTD, while heat intensifies these effects. Basic conditions, especially at pH = 11, also alter structures, particularly in the S2D. The data underscore the S-protein’s differential response to acidic and basic environments, with extreme pH conditions dominate the structure impact while, at moderate pH, thermal effects lead.

This work highlights the potential impact of environmental factors on the structures of the S-protein and these insights could be critical in forming antiviral strategies, in devising storage conditions for vaccines, and even in designing therapeutics. Further studies, leveraging on potential advances in modeling technologies, could explore the longer-term effects at extreme pH and thermal conditions on the S-protein and, even, the entire virus.

## Data Availability

The datasets presented in this study can be found in online repositories. The names of the repository/repositories and accession number(s) can be found below: https://github.com/Niuzyx/TpH-Sprotein-simulation.git, The data that support the findings of this study are available in github.
