# Supplementary material for: Simulations of pH and thermal effects on SARS-CoV-2 spike glycoprotein
Source: Front Mol Biosci. 2025 Feb 11;12:1545041. doi: 10.3389/fmolb.2025.1545041 (PMC11850259; doi:10.3389/fmolb.2025.1545041)
Supplement: Supplementary file 1 [file DataSheet1.pdf]

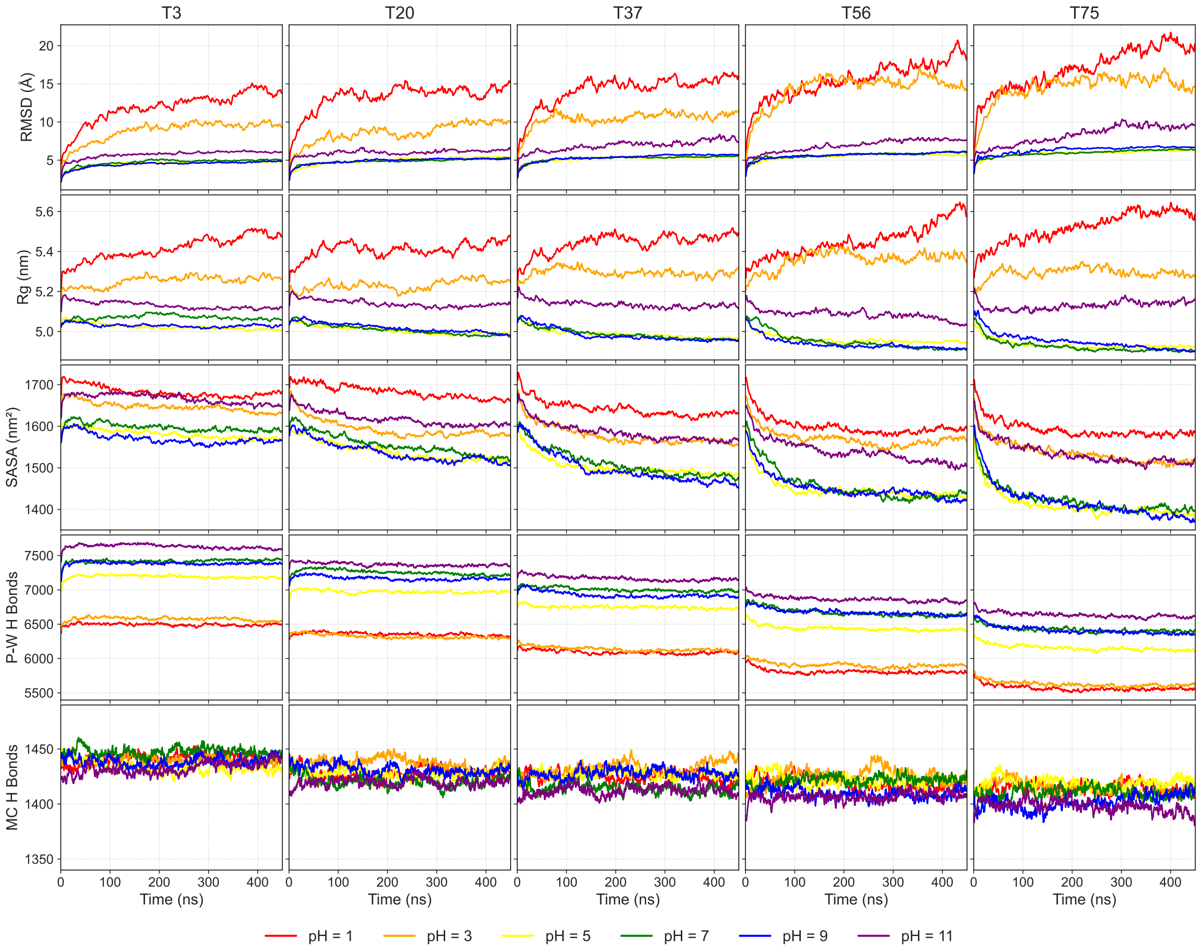

Fig. S1 The time series for protein measurements

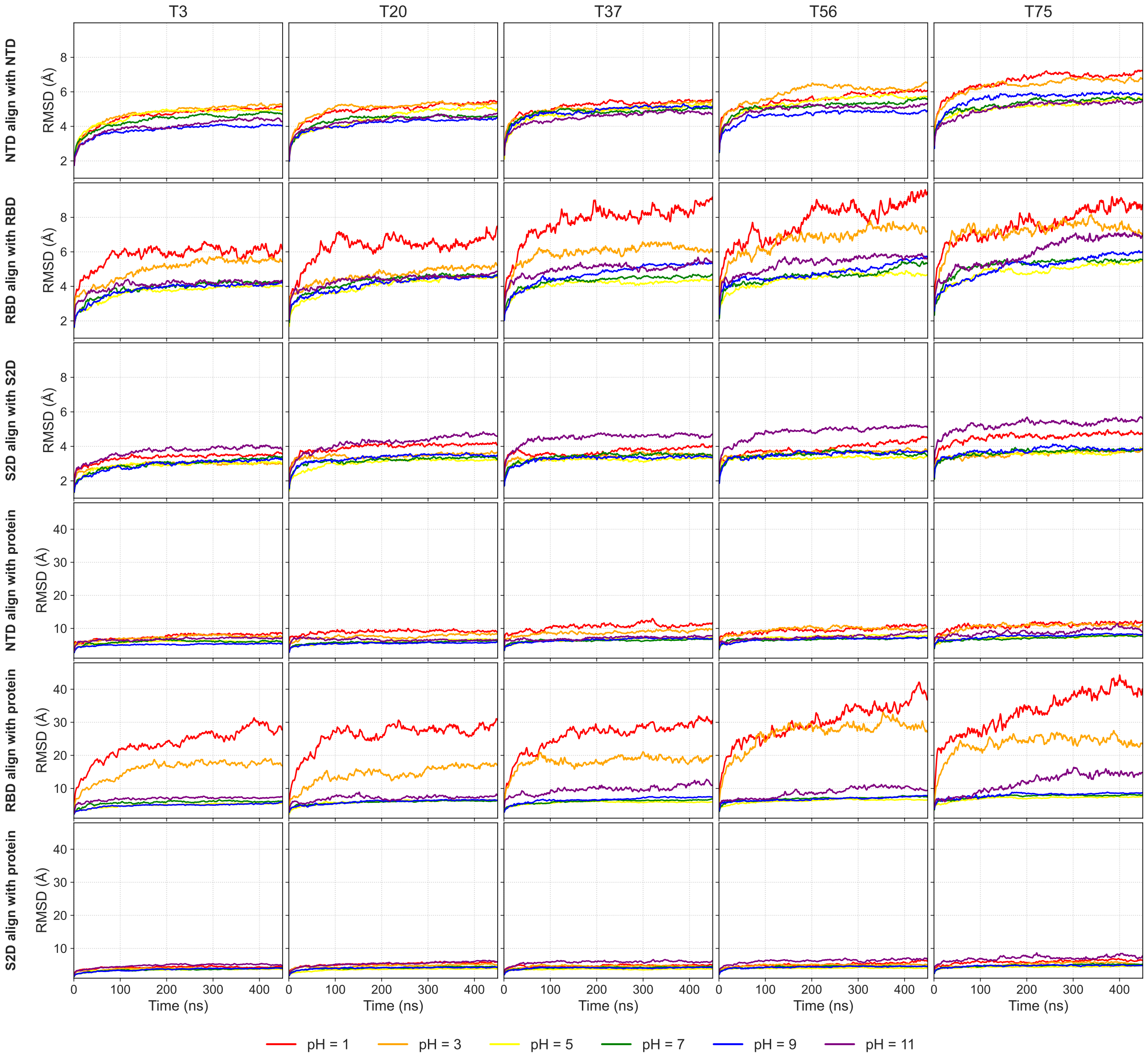

Fig. S2 The time series for domain-based RMSD

Table S1. The means and standard errors of the protein measurements.

Temp.	pH	RMSD (Å)	Rg (nm)	SASA (nm^2)	IA (nm^2)	# P-W H bonds	# MC H bonds
3˚C	1	13.5 ± 1.5	5.5 ± 0.1	1674.0 ± 14.8	29.8 ± 0.1	6487.7 ± 22.7	1443.7 ± 14.4
	3	9.7 ± 1.1	5.3 ± 0.0	1639.1 ± 3.6	33.4 ± 0.5	6566.7 ± 20.1	1439.1 ± 9.4
	5	4.8 ± 0.0	5.0 ± 0.0	1572.1 ± 6.5	41.8 ± 0.6	7180.8 ± 20.5	1431.8 ± 1.8
	7	5.0 ± 0.2	5.1 ± 0.0	1590.1 ± 8.0	39.3 ± 0.3	7432.4 ± 11.1	1447.9 ± 1.1
	9	4.7 ± 0.2	5.0 ± 0.0	1561.4 ± 12.6	39.3 ± 0.4	7381.0 ± 17.9	1440.2 ± 3.5
	11	6.1 ± 0.5	5.1 ± 0.0	1655.3 ± 10.6	32.0 ± 0.3	7610.4 ± 16.2	1436.8 ± 9.1
20˚C	1	14.1 ± 1.6	5.4 ± 0.0	1669.0 ± 10.5	29.5 ± 0.9	6341.5 ± 6.1	1430.0 ± 2.7
	3	9.5 ± 1.4	5.2 ± 0.1	1581.4 ± 10.7	35.8 ± 1.1	6304.2 ± 33.9	1434.5 ± 6.7
	5	5.3 ± 0.2	5.0 ± 0.0	1520.9 ± 2.0	42.9 ± 0.2	6966.7 ± 14.5	1427.5 ± 2.8
	7	5.1 ± 0.1	5.0 ± 0.0	1534.4 ± 7.4	41.5 ± 0.2	7228.8 ± 15.9	1419.5 ± 4.9
	9	5.2 ± 0.2	5.0 ± 0.0	1518.2 ± 2.0	42.6 ± 0.5	7154.2 ± 28.4	1429.2 ± 5.7
	11	6.2 ± 0.2	5.1 ± 0.0	1603.7 ± 9.6	35.1 ± 0.9	7353.9 ± 26.6	1420.0 ± 7.2
37˚C	1	15.3 ± 2.1	5.5 ± 0.1	1627.9 ± 21.1	31.2 ± 0.8	6079.9 ± 29.8	1424.5 ± 3.9
	3	10.9 ± 0.2	5.3 ± 0.1	1563.5 ± 7.3	34.0 ± 1.1	6120.9 ± 19.2	1433.6 ± 2.8
	5	5.4 ± 0.2	5.0 ± 0.0	1489.2 ± 10.0	45.5 ± 0.9	6739.1 ± 10.2	1425.8 ± 6.4
	7	5.4 ± 0.1	5.0 ± 0.0	1483.9 ± 11.6	45.7 ± 0.1	6986.5 ± 30.2	1412.7 ± 3.9
	9	5.7 ± 0.1	5.0 ± 0.0	1470.8 ± 8.0	46.2 ± 0.4	6907.9 ± 4.2	1428.1 ± 2.0
	11	7.4 ± 1.1	5.1 ± 0.0	1568.5 ± 7.8	37.1 ± 0.2	7140.5 ± 3.5	1413.5 ± 5.1
56˚C	1	17.5 ± 3.2	5.5 ± 0.1	1588.2 ± 5.8	31.7 ± 0.7	5798.9 ± 15.1	1416.0 ± 4.2
	3	15.2 ± 2.2	5.4 ± 0.1	1558.9 ± 9.1	35.4 ± 0.8	5883.9 ± 25.5	1426.9 ± 5.2
	5	5.7 ± 0.4	5.0 ± 0.0	1435.9 ± 10.0	47.4 ± 0.9	6416.9 ± 6.0	1420.7 ± 4.2
	7	5.9 ± 0.1	4.9 ± 0.0	1430.8 ± 11.8	49.6 ± 0.8	6632.5 ± 27.5	1423.0 ± 5.0
	9	5.9 ± 0.1	4.9 ± 0.0	1434.5 ± 2.1	47.5 ± 0.4	6645.2 ± 11.8	1408.2 ± 5.7
	11	7.6 ± 0.8	5.0 ± 0.0	1519.2 ± 11.9	39.2 ± 0.9	6843.2 ± 7.8	1407.6 ± 2.9
75˚C	1	19.5 ± 0.6	5.6 ± 0.0	1581.2 ± 9.5	31.7 ± 0.6	5554.2 ± 37.7	1414.3 ± 7.6
	3	15.4 ± 2.0	5.3 ± 0.0	1516.3 ± 4.9	35.4 ± 0.8	5605.0 ± 15.0	1411.7 ± 4.6
	5	6.2 ± 0.2	4.9 ± 0.0	1393.7 ± 5.9	47.4 ± 0.5	6132.7 ± 15.7	1418.5 ± 9.4
	7	6.3 ± 0.1	4.9 ± 0.0	1401.3 ± 4.5	49.6 ± 0.6	6398.5 ± 25.1	1409.9 ± 1.7
	9	6.6 ± 0.2	4.9 ± 0.0	1390.5 ± 18.8	47.5 ± 2.2	6374.8 ± 27.0	1406.1 ± 2.8
	11	9.4 ± 2.4	5.1 ± 0.1	1514.8 ± 10.7	39.2 ± 0.8	6609.5 ± 16.0	1394.9 ± 2.9

Table S2. Mean and standard error of secondary structure components.

Temp.	pH	α-helix	β-Sheet	β-bridge	3_{10}-helix	π-helix	Turn	SS	Coil	Bend
3˚C	1	755 ± 8	911 ± 14	37 ± 2	86 ± 3	17 ± 2	306 ± 15	61%	928 ± 11	446 ± 4
	3	743 ± 8	908 ± 11	37 ± 1	84 ± 11	14 ± 2	300 ± 6	60%	935 ± 4	465 ± 3
	5	721 ± 9	914 ± 5	40 ± 2	79 ± 9	17 ± 3	306 ± 1	60%	943 ± 4	466 ± 9
	7	733 ± 9	944 ± 3	30 ± 1	84 ± 3	16 ± 1	337 ± 11	61%	905 ± 4	437 ± 6
	9	748 ± 6	927 ± 9	33 ± 2	76 ± 6	12 ± 2	336 ± 7	61%	916 ± 9	438 ± 4
	11	758 ± 7	937 ± 7	28 ± 2	59 ± 3	25 ± 0	317 ± 8	61%	927 ± 7	435 ± 9
20˚C	1	717 ± 1	916 ± 3	45 ± 4	96 ± 4	15 ± 0	307 ± 8	60%	956 ± 16	434 ± 12
	3	734 ± 12	921 ± 5	42 ± 2	96 ± 8	14 ± 4	313 ± 7	61%	923 ± 11	443 ± 4
	5	718 ± 8	913 ± 6	43 ± 1	71 ± 9	12 ± 2	322 ± 9	60%	938 ± 6	469 ± 12
	7	697 ± 10	925 ± 13	35 ± 3	85 ± 7	18 ± 2	330 ± 4	60%	956 ± 15	440 ± 7
	9	729 ± 2	939 ± 4	28 ± 1	86 ± 13	18 ± 1	311 ± 8	61%	921 ± 11	454 ± 4
	11	718 ± 6	955 ± 1	28 ± 0	69 ± 5	16 ± 0	321 ± 10	60%	926 ± 11	453 ± 8
37˚C	1	725 ± 7	915 ± 10	36 ± 5	78 ± 6	16 ± 1	326 ± 2	60%	983 ± 9	407 ± 12
	3	741 ± 6	918 ± 1	42 ± 3	86 ± 5	19 ± 1	308 ± 7	61%	963 ± 8	409 ± 6
	5	712 ± 5	917 ± 4	46 ± 2	78 ± 2	13 ± 4	329 ± 8	60%	953 ± 10	438 ± 4
	7	723 ± 5	932 ± 12	30 ± 3	67 ± 4	16 ± 0	329 ± 7	60%	943 ± 16	446 ± 3
	9	727 ± 11	942 ± 15	39 ± 3	72 ± 6	21 ± 0	332 ± 9	61%	927 ± 5	426 ± 11
	11	715 ± 6	954 ± 5	31 ± 2	59 ± 7	17 ± 2	324 ± 4	60%	948 ± 12	438 ± 6
56˚C	1	708 ± 2	924 ± 4	44 ± 2	97 ± 14	15 ± 0	292 ± 12	60%	986 ± 15	420 ± 9
	3	730 ± 8	917 ± 8	45 ± 4	79 ± 10	15 ± 0	315 ± 16	60%	991 ± 18	394 ± 3
	5	693 ± 7	921 ± 11	45 ± 4	73 ± 5	20 ± 2	348 ± 9	60%	951 ± 4	435 ± 9
	7	724 ± 6	934 ± 5	36 ± 6	65 ± 4	18 ± 2	333 ± 1	61%	949 ± 15	427 ± 8
	9	719 ± 8	923 ± 14	39 ± 2	60 ± 7	17 ± 2	303 ± 4	59%	980 ± 18	445 ± 14
	11	705 ± 3	963 ± 14	29 ± 2	70 ± 7	18 ± 2	326 ± 3	61%	955 ± 14	420 ± 1
75˚C	1	714 ± 3	959 ± 3	47 ± 4	91 ± 4	14 ± 2	295 ± 7	61%	977 ± 8	389 ± 2
	3	728 ± 10	922 ± 12	48 ± 4	71 ± 2	15 ± 0	312 ± 16	60%	989 ± 26	401 ± 12
	5	701 ± 9	929 ± 8	44 ± 3	69 ± 4	16 ± 1	339 ± 3	60%	980 ± 15	408 ± 6
	7	691 ± 7	944 ± 8	42 ± 1	70 ± 5	19 ± 2	328 ± 6	60%	963 ± 10	429 ± 1
	9	710 ± 7	941 ± 8	36 ± 3	67 ± 6	16 ± 0	318 ± 8	60%	977 ± 4	421 ± 8
	11	702 ± 10	965 ± 4	26 ± 2	51 ± 12	17 ± 2	323 ± 10	60%	1003 ± 7	399 ± 8

Table S3. The means and standard errors of the domain measurements.

Temp.	pH	RMSD (Å) Align with Domain			RMSD (Å) Align with Protein		
		NTD	RBD	S2D	NTD	RBD	S2D
3˚C	1	5.0 ± 0.1	6.1 ± 0.3	3.5 ± 0.1	8.2 ± 0.3	27.3 ± 1.8	4.4 ± 0.2
	3	5.2 ± 0.1	5.5 ± 0.1	3.0 ± 0.1	7.7 ± 0.3	17.7 ± 0.6	4.0 ± 0.1
	5	4.9 ± 0.1	4.0 ± 0.1	3.0 ± 0.1	6.1 ± 0.1	5.3 ± 0.1	3.7 ± 0.1
	7	4.7 ± 0.1	4.2 ± 0.1	3.2 ± 0.1	6.0 ± 0.2	6.1 ± 0.2	3.8 ± 0.2
	9	4.0 ± 0.1	4.1 ± 0.1	3.2 ± 0.1	5.3 ± 0.1	5.2 ± 0.2	3.9 ± 0.1
	11	4.3 ± 0.1	4.2 ± 0.1	3.9 ± 0.1	7.1 ± 0.2	7.2 ± 0.2	5.1 ± 0.2
20˚C	1	5.3 ± 0.1	6.5 ± 0.3	4.1 ± 0.1	9.2 ± 0.3	28.0 ± 1.1	5.7 ± 0.1
	3	5.3 ± 0.1	5.0 ± 0.2	3.5 ± 0.1	8.0 ± 0.5	16.1 ± 1.2	5.0 ± 0.2
	5	5.0 ± 0.1	4.5 ± 0.1	3.2 ± 0.0	6.5 ± 0.2	6.4 ± 0.2	3.8 ± 0.1
	7	4.6 ± 0.1	4.6 ± 0.1	3.3 ± 0.1	6.0 ± 0.2	6.2 ± 0.1	4.4 ± 0.2
	9	4.4 ± 0.1	4.5 ± 0.1	3.5 ± 0.1	5.6 ± 0.1	6.4 ± 0.2	4.2 ± 0.1
	11	4.5 ± 0.1	4.6 ± 0.1	4.5 ± 0.2	6.4 ± 0.2	7.4 ± 0.3	5.8 ± 0.3
37˚C	1	5.4 ± 0.1	8.3 ± 0.4	3.9 ± 0.1	10.1 ± 0.7	28.8 ± 1.5	4.9 ± 0.2
	3	5.2 ± 0.1	6.2 ± 0.2	3.5 ± 0.1	9.2 ± 0.4	19.1 ± 0.7	4.7 ± 0.2
	5	5.1 ± 0.1	4.3 ± 0.1	3.2 ± 0.1	7.0 ± 0.1	5.8 ± 0.1	3.7 ± 0.1
	7	4.9 ± 0.1	4.5 ± 0.1	3.5 ± 0.1	6.5 ± 0.2	6.4 ± 0.1	4.3 ± 0.1
	9	5.1 ± 0.1	5.2 ± 0.1	3.3 ± 0.1	7.0 ± 0.1	7.2 ± 0.2	4.1 ± 0.1
	11	4.8 ± 0.1	5.1 ± 0.2	4.6 ± 0.1	7.4 ± 0.2	10.5 ± 0.1	6.0 ± 0.2
56˚C	1	5.9 ± 0.1	8.6 ± 0.5	4.2 ± 0.1	10.2 ± 0.7	35.0 ± 2.6	5.6 ± 0.4
	3	6.2 ± 0.1	7.3 ± 0.3	3.7 ± 0.1	10.0 ± 0.4	29.1 ± 1.4	5.0 ± 0.1
	5	5.7 ± 0.1	4.7 ± 0.1	3.3 ± 0.1	7.8 ± 0.2	6.6 ± 0.2	4.1 ± 0.1
	7	5.3 ± 0.1	4.9 ± 0.3	3.6 ± 0.1	7.0 ± 0.2	7.3 ± 0.2	4.6 ± 0.1
	9	4.8 ± 0.1	5.2 ± 0.3	3.6 ± 0.1	7.0 ± 0.2	7.3 ± 0.3	4.5 ± 0.1
	11	5.1 ± 0.1	5.7 ± 0.1	5.1 ± 0.1	8.0 ± 0.6	10.1 ± 0.6	6.5 ± 0.3
75˚C	1	7.0 ± 0.1	8.3 ± 0.5	4.7 ± 0.1	11.5 ± 0.5	39.0 ± 2.2	6.4 ± 0.3
	3	6.7 ± 0.1	7.5 ± 0.3	3.7 ± 0.1	11.1 ± 0.4	24.8 ± 1.3	5.7 ± 0.2
	5	5.4 ± 0.2	5.2 ± 0.1	3.6 ± 0.2	7.6 ± 0.3	7.3 ± 0.1	4.5 ± 0.1
	7	5.5 ± 0.1	5.5 ± 0.1	3.8 ± 0.1	7.6 ± 0.3	7.9 ± 0.1	5.1 ± 0.1
	9	5.9 ± 0.1	5.7 ± 0.2	3.9 ± 0.1	8.1 ± 0.3	8.4 ± 0.2	4.8 ± 0.1
	11	5.4 ± 0.1	6.8 ± 0.2	5.4 ± 0.1	9.6 ± 0.7	14.4 ± 0.8	7.3 ± 0.5

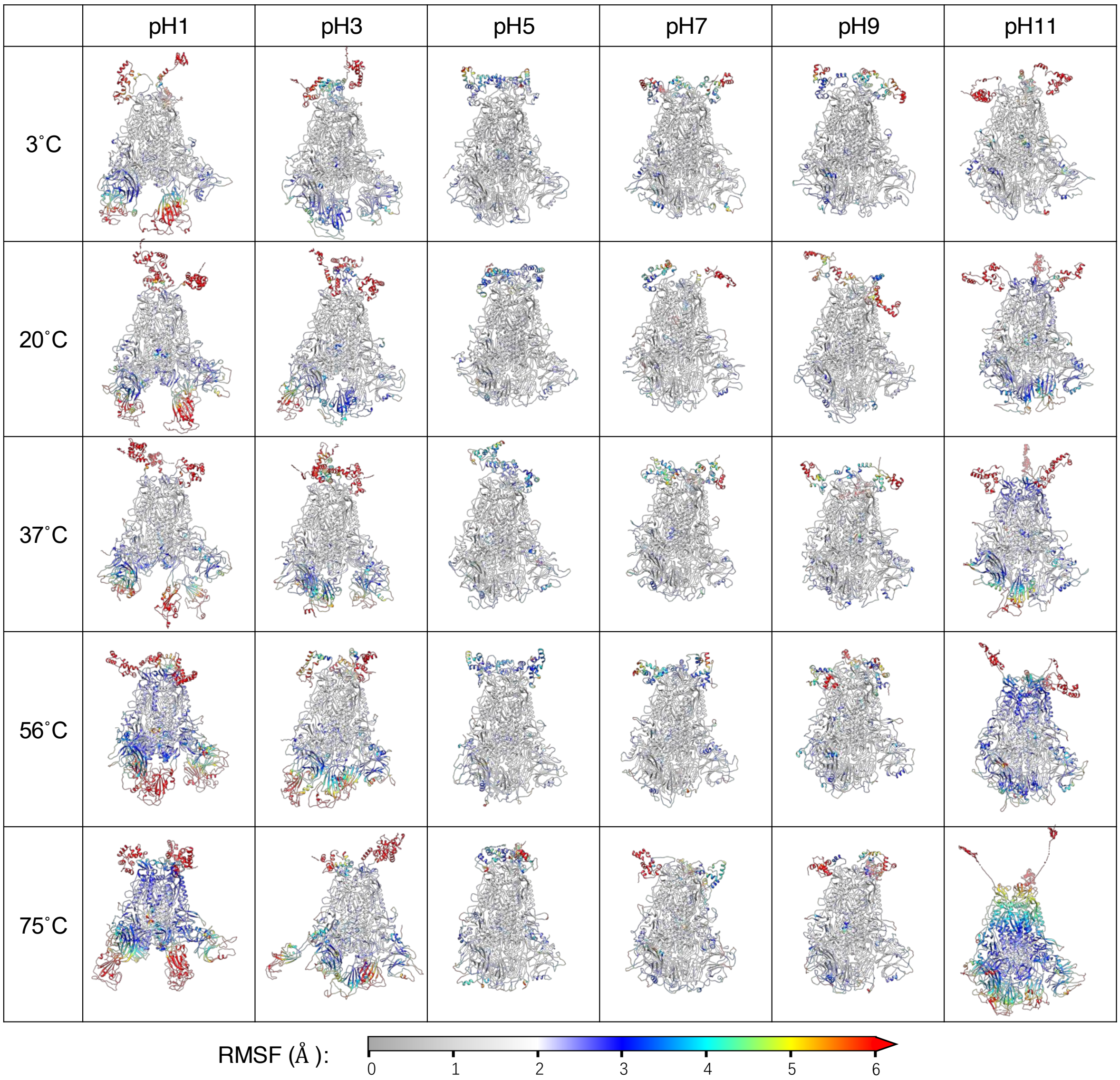

Fig. S3 The averaged structure with each residue colored by the RMSF for all conditions.
